# Cytokines and microRNAs in SARS-CoV-2: What do we know?

**DOI:** 10.1016/j.omtn.2022.06.017

**Published:** 2022-06-25

**Authors:** Fahimeh Zamani Rarani, Bahman Rashidi, Mohammad Hassan Jafari Najaf Abadi, Michael R. Hamblin, Seyed Mohammad Reza Hashemian, Hamed Mirzaei

**Affiliations:** 1 Department of Anatomical Sciences, School of Medicine, Isfahan University of Medical Sciences, Isfahan, Iran; 2 Department of Medical Biotechnology, School of Medicine, Mashhad University of Medical Sciences, Mashhad, Iran; 3 Laser Research Centre, Faculty of Health Science, University of Johannesburg, Doornfontein 2028, South Africa; 4 Chronic Respiratory Diseases Research Center (CRDRC), National Research Institute of Tuberculosis and Lung Diseases (NRITLD), Shahid Beheshti University of Medical Sciences, Tehran, Iran; 5 Student Research Committee, Kashan University of Medical Sciences, Kashan, Iran; 6 Research Center for Biochemistry and Nutrition in Metabolic Diseases, Institute for Basic Sciences, Kashan University of Medical Sciences, Kashan, IR, Iran

**Keywords:** SARS-CoV-2, COVID-19, microRNAs, cytokines, interferons, interleukins, NF-κB, Janus kinases, STAT transcription factors

## Abstract

The coronavirus disease 2019 (COVID-19) pandemic constitutes a global health emergency. Currently, there are no completely effective therapeutic medications for the management of this outbreak. The cytokine storm is a hyperinflammatory medical condition due to excessive and uncontrolled release of pro-inflammatory cytokines in patients suffering from severe COVID-19, leading to the development of acute respiratory distress syndrome (ARDS) and multiple organ dysfunction syndrome (MODS) and even mortality. Understanding the pathophysiology of COVID-19 can be helpful for the treatment of patients. Evidence suggests that the levels of tumor necrosis factor alpha (TNF-α) and interleukin (IL)-1 and IL-6 are dramatically different between mild and severe patients, so they may be important contributors to the cytokine storm. Several serum markers can be predictors for the cytokine storm. This review discusses the cytokines involved in severe acute respiratory syndrome coronavirus 2 (SARS-CoV-2) infection, focusing on interferons (IFNs) and ILs, and whether they can be used in COVID-19 treatment. Moreover, we highlight several microRNAs that are involved in these cytokines and their role in the cytokine storm caused by COVID-19.

## Graphical abstract

**Figure:**
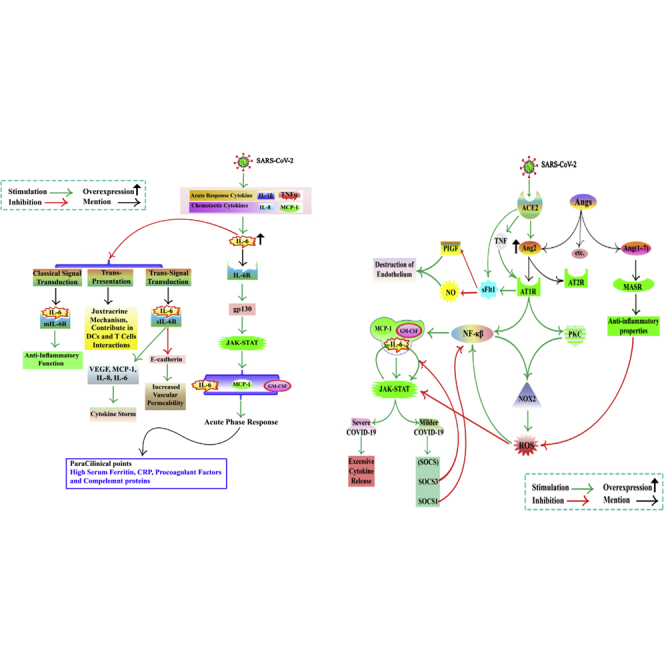

## Teaser

Our research indicated discrepancies in the reported results related to the impact of many cytokines on the treatment of COVID-19, and some studies reported that the disease phase is very important in the use of cytokines and will have different effects. Moreover, we suggest that cytokine-related microRNAs may be a promising target in treating COVID-19.

## Introduction

The local outbreak of coronavirus disease 2019 (COVID-19) in December 2019 was recognized for the first time in Wuhan, China. The disease spread rapidly to all parts of the world and, on March 11, the World Health Organization (WHO) designated this outbreak as a global pandemic.^1^^,^^2^

The infectious disease COVID-19 is caused by a new coronavirus (CoV), called severe acute respiratory syndrome coronavirus 2 (SARS-CoV-2). The CoVs are a family of positive single-stranded (+ss) RNA viruses covered by an envelope, containing four genera based on nucleic acid and protein sequence: alpha CoVs, beta CoVs, gamma CoVs, and delta CoVs.^2^^,^^3^ Examples of beta-CoVs are Middle East respiratory syndrome coronavirus (MERS-CoV), severe acute respiratory syndrome coronavirus (SARS-CoV), human coronavirus HKU1 (HCoV-HKU1), human coronavirus OC43 (HCoV-OC43), and human coronavirus 229E (HCoV-229E).^3^^,^^4^ From 2002 up to now, three global disease outbreaks have been shown to be caused by beta-CoVs that affect the airways, especially the lower tract, which were SARS-CoV in 2002, MERS-CoV in 2012, and SARS-CoV-2 from 2019. These infections resulted in multiple organ dysfunction syndrome (MODS), acute respiratory distress syndrome (ARDS), and even mortality.^5^^,^^6^

COVID-19 remains a health issue of great concern throughout the world. Fever, non-productive cough, fatigue, myalgia, rhinorrhea, pharyngalgia, diarrhea, dyspnea, and hypoxemia are the most frequently reported symptoms.^7^^,^^8^ Some cases have no or only mild symptoms, but others require hospitalization and intensive care. It has been found that pneumonia and respiratory failure are the leading causes of death, and death rates are very high in patients who require mechanical ventilation.^9^^,^^10^

Many studies have reported that the spike (S) protein of SARS-CoV-2 mediates its entry into the targeted human cells, such as airway epithelial cells, and olfactory receptor neurons. This may be the starting point of a process that will eventually lead to ARDS, MODS, and death. Viruses can multiply and spread rapidly to other target cells and organs. It is reported that the virus can affect a wide range of cells by direct attachment to the receptor or indirectly via cytokine production.^11^, ^12^, ^13^

Cytokines are cell signaling molecules composed of proteins, glycoproteins, or peptides. They are secreted by numerous cell types and play a crucial role in many biological functions by binding to cell surface receptors at particular concentrations. Cytokines exert either pro-inflammatory or anti-inflammatory effects, and inflammation is associated with an imbalance between anti-inflammatory and pro-inflammatory cytokines.^14^, ^15^, ^16^

In severe COVID-19, the virus activates both the innate and adaptive immune systems, resulting in uncontrolled inflammatory responses and overproduction of cytokines, finally producing a cytokine storm. A cytokine storm is a medical condition in which excessive and uncontrolled levels of pro-inflammatory cytokines are produced in response to different triggers, and then continue to be produced in a destructive auto-amplifying cycle typical of MOD.^17^^,^^18^ In COVID-19 pathogenesis, cells undergo apoptosis or necrosis induced directly or indirectly by cytokines. The cytokines can bind to death receptors or activate other cells such as natural killer (NK) cells. The breach of the blood-airway barrier caused by the cytokine storm leads to pulmonary edema and respiratory failure. It also permits the penetration of SARS-CoV-2 into the systemic circulation, where it can then cause systemic MOD.^17^^,^^19^^,^^20^ Contradictory results have been found regarding the modulation of cytokines to manage the disease. Some researchers believe that there are insufficient studies supporting the benefits of cytokine gene regulation in reducing the mortality rate in COVID-19.^18^^,^^21^

MicroRNAs (miRNAs, or miR) or short non-coding single-stranded RNAs, are able to regulate the expression of one-third of genes in human beings.^22^^,^^23^ It has been shown that miRNAs can affect many signaling pathways and regulate cytokine production. Cytokines can affect miRNA expression by inducing transcription factor miRNAs.^23^^,^^24^

Collectively, the cytokine profiles in COVID-19 are very complex, and the role of cytokine dysregulation induced by SARS-CoV-2 needs more clarification. Our goal in this article is to provide researchers and physicians a comprehensive overview of cytokine-related effects in COVID-19 up to the present.

### Cytokines

Cytokines (in Greek, cyto means cell, and kinos means movement) are small proteins, glycoproteins, or signaling peptides produced and released by various cell types. They have several biological functions at various concentrations by attaching to specific cell receptors.^14^^,^^25^ Based on their biological effects, the cytokine superfamily can be divided into six main families: interleukins (ILs), interferons (IFNs), tumor necrosis factors (TNFs), hematopoietic growth factors, chemokines, and transforming growth factor (TGF) β (TGF-β). Cytokines can also be divided into pro-inflammatory or anti-inflammatory sub-classes.^15^^,^^26^

Cytokines can mediate cell-cell interactions and communication. They may act in an autocrine, paracrine, or endocrine manner, and can exhibit pleiotropic effects. Various cell types may secrete the same cytokine, or be affected by a single cytokine. Often, one cytokine acts on certain cells to produce additional cytokines, forming a cytokine cascade.^15^

There are numerous reports of abnormal cytokine concentrations in patients during COVID-19 infection. One critical process in severe COVID-19 patients is that excessive rates of inflammatory cytokine production and dysregulated gene expression in affected cells results in a cytokine storm. One of the leading reasons for multiple organ failure (MOF) and ARDS is the cytokine storm.^27^, ^28^, ^29^ Therefore, it is necessary to understand the cytokines involved in COVID-19 that have been studied so far.

### IFNs

IFNs are a group of soluble glycoproteins. They provide the most critical first line of defense against pathogenic agents like viruses.^30^ According to their protein structure and receptors, IFNs are categorized into three classes: type I IFNs (IFN subtypes alpha, beta, epsilon, kappa, and omega), type II IFNs (IFN-γ), and type III IFNs ( IFN-λ).^31^^,^^32^

### Type I IFNs

The type I IFNs in mammalian species include alpha (IFN-α), beta (IFN-β), kappa (IFN-κ), delta (IFN-δ), epsilon (IFN-ε), tau (IFN-τ), omega (IFN-ω), and zeta (IFN-ζ, also called limitin).^25^^,^^33^ They all have a specific type I IFN cell surface receptor (IFNAR) in target cells and act in an autocrine and/or paracrine manner. The Janus kinase-signal transducer and activator of transcription (JAK-STAT) signaling pathway and nuclear factor κB NF-κB) pathway are mainly involved in type I IFN functions.^34^, ^35^, ^36^, ^37^, ^38^

When viral infection occurs and the body recognizes it, type I IFNs are produced. Different cell types can secrete type I IFNs. This stimulates a powerful antiviral defense response involving numerous IFN-stimulated genes (ISGs) designed to interfere with the replication and pathogenesis of the virus.^30^^,^^33^ Reportedly, the new CoV, SARS-CoV-2, is more sensitive to type I IFNs compared with SARS-CoV.^39^ Nevertheless, in COVID-19, it has been reported that the weak type I IFN response in patients suffering from severe hyperinflammation driven by NF-κB correlates with negligible clearance of the virus. Studies on the efficacy of type I IFN therapy in COVID-19 patients were disappointing.^25^^,^^40^

### IFN-α

IFN-α is produced by a wide range of cells, such as respiratory epithelial cells, macrophages, and dendritic cells (DCs). It is known that many types of cells, especially peripheral blood B cells and monocytes, are IFN-α targets.^41^^,^^42^ Studies have shown a low level of IFN-α in the blood of hospitalized COVID-19 patients that go on to develop severe/life-threatening COVID-19.^43^ It has been shown that IFN-α plays a pivotal role in early-stage COVID-19. IFN-α can reduce the number of viruses, resulting in fewer symptoms and a shorter disease duration. IFN-α administration has been helpful in the management of some viral diseases like SARS. It can also induce angiotensin-converting enzyme (ACE) 2 as an IFN-stimulated gene (ISG) in human upper airway epithelial cells.^43^, ^44^, ^45^

A clinical trial found that IFN-α2b administration in the early phase of COVID-19 could decrease the mortality rate, while late use of IFN-α2b could actually increase mortality. Therefore, IFN-α2b administration in early-stage COVID-19 may produce promising results.^46^

### IFN-β

IFN-β is secreted by pulmonary epithelial cells and monocyte-derived cells.^41^ IFN-β activates B cells, T cells, monocytes, macrophages, and DCs.^47^

Open reading frame (ORF) 6, ORF8, and nucleocapsid proteins related to SARS-CoV-2 are potent inhibitors of IFN-β and NF-κB-responsive gene promoters. In Sendai virus infection, these proteins inhibited the IFN-stimulated response element (ISRE), and ORF6 and ORF8 proteins after IFN-β treatment could block the ISRE.^48^ In critical COVID-19 patients, highly impaired production and response of IFN-β has been found, and the viral load remains high. In these patients, severe inflammatory responses have been observed.^49^

IFN-β can also induce useful antiviral activity via ISGs, but ISGs are significantly inhibited in SARS-CoV-2 infection. In early-stage COVID-19 infection, non-structural proteins (NSPs) and ORFs related to the virus block host IFNs and dysregulate the ISGs, while ISGs with pro-inflammatory potential are stimulated during the later phase of SARS-CoV-2 infection.^50^^,^^51^

It was previously reported that IFN-β-1b administration in severe COVID-19 patients was associated with positive clinical improvement and shorter duration of hospital stay without serious adverse effects in patients.^52^ IFN-β can block the overexpression of IL-6 and IL-8 and enhance immune response. Studies have reported that glucocorticoids could inhibit IFN-β signaling and many other cytokines in the human lungs, so care must be taken when using glucocorticoids in viral-induced ARDS.^53^^,^^54^

### IFN-κ

IFN-κ affects innate immune-related cells and can be used to control systemic or local immune responses.^55^ It is produced by monocyte-derived macrophages, DCs, and keratinocytes. IFN-κ affects target cells such as monocytes and DCs. It has been shown that inhalation of IFN-κ plus Trefoil factor 2 (TFF2) can promote the repair of airway epithelial cells exposed to injury, and significantly improves symptoms such as cough in asthma patients. Use of this combination in COVID-19 treatment may significantly improve clinical symptoms, virus negativity, and computed tomography (CT) scan results, and reduce the duration of hospitalization.^31^^,^^56^^,^^57^

### IFN-δ

IFN-δ is secreted by porcine blastocysts. It is reported that IFN-δ has immunomodulatory and antiviral activity via type I IFNs, but its antiviral activity is lower than IFN-α.^58^^,^^59^ Few relevant studies are available for the effects of IFN-δ.

### IFN-ε

IFN-ε is expressed in the lung, brain, skin, intestinal tract, and reproductive tissues (uterus, cervix, vagina, and ovary). IFN-ε contributes to antiviral and antibacterial mucosal immunity. It has been reported that IFN-ε can suppress human immunodeficiency virus (HIV) replication.^60^, ^61^, ^62^

The IFN-ε ligand can bind to IFNAR1 and IFNAR2 and affect the JAK-STAT pathway. These pathways regulate mucosal immune responses resulting in protection from viral and bacterial invasion.^63^ In some mammalian species, such as pangolins, which are a potential natural reservoir of CoVs, IFN-ε is produced by epithelial cells and contributes to mucosal immunity in skin, lung, intestine, and reproductive tissues.^64^

Some COVID-19 patients reported changes in skin pigmentation, rash, and low spermatogenesis rate. IFN-ε is expressed in both tissues (skin and testes), so it is possible that COVID-19 infection is an androgen-mediated process.^65^, ^66^, ^67^ IFN-ε protects the reproductive system in women from HIV-1 and other viral infections, and is regulated by endogenous and exogenous hormones. Therefore IFN-ε may be an explanation of decreased mortality in women with SARS-CoV-2 infection.^63^^,^^68^

### IFN-τ

IFN-τ is released by bovine blastocysts.^69^ Although IFN-τ is not expressed in humans, human and mouse cells, such as monocyte-derived macrophages, are able to respond to its administration. IFN-τ has antiviral and antitumor effects, and binds to IFN-α receptors. It induces intracellular signaling leading to antiviral cytokine production, such as IL-4 and IL-6.^70^ IFN-τ reduces the replication of various viruses, including human papillomavirus (HPV), feline immunodeficiency virus (FIV), and HIV.^71^ There is no report of its effect on SARS-CoV-2. It has been reported that administration of IFN-τ in a mouse allergy model decreased cell infiltration into lung tissue under inflammatory conditions.^70^

### IFN-ω

DCs can produce IFN-ω in response to viral infection.^72^ Leukocytes and epithelial cells may be target cells for IFN-ω.^73^ It stimulates a signaling pathway similar to the IFN-α/β receptor (IFNAR). It has also been reported that the activation of phosphoinositide-3-kinase/protein kinase B (P13K/Akt) signaling is required for IFN-ω activity.^74^^,^^75^ It was shown that IFN-ω had an anti-SARS activity similar to IFN-β. IFN-ω administration reduced the severity of the disease and inhibited CoV replication in monkeys. A study reported that some patients with severe COVID-19-mediated pneumonia showed neutralizing immunoglobulin (Ig) G against IFN-ω.^76^

### IFN-ζ

IFN-ζ is only found in mice, and is secreted by the salivary duct and bronchial epithelial cells. It has sequence homology with IFN-α, and shows similar antiviral and immunomodulatory effects. IFN-ζ has no or low lympho-myelo-suppressive activity. It has been suggested that IFN-α/βR could be a receptor for IFN-ζ. It was shown that the IRF-1 pathway in fibroblasts was involved with IFN-ζ activity and antiviral functions.^77^, ^78^, ^79^ To date, no research has been done on its effects in COVID-19.

### Type II IFNs (IFN-γ)

IFN-γ is secreted by macrophages, T cells, and NK cells. The primary source of IFN-γ in rhinovirus infections was bronchial epithelial cells. Its receptors are expressed on immune cells like T cells and NK cells. Similar to type I IFN, IFN-γ can stimulate the JAK-STAT signaling pathway and the NF-κB pathway. IFN-γ can act through the activation of JAK1, JAK2, and STAT signaling pathways.^80^^,^^81^

IFN-γ contributes to the inhibition of acute inflammation, and the transition from innate to acquired immunity. It was reported that the interplay of IFN-γ with IL-6/sIL-6R signaling had positive effects on neutrophil recruitment and phagocytosis. Also, it was observed that patients suffering from severe COVID-19 exhibited a larger IL-6/IFN-γ ratio than moderate cases. IFN-γ may enhance the cytokine storm, leading to lung failure. By contrast, another study reported that IFN-γ production tended to be lower in the severe forms of COVID-19 than in the moderate cases.^82^^,^^83^ It seems that more studies are needed.

### Type III IFNs (IFN-λ)

IFN-λ is secreted by epithelial cells, monocyte-derived macrophages, NK cells, dendritic cells (DCs), cytotoxic T cells, and regulatory T cells. There are four sub-classes of IFN-λ: IFN-λ1 (or IL-29), IFN-λ2 (or IL-28A), IFN-λ3 (or IL-28B), and IFN-λ4. These can bind to a complex resulting in IL-10R2 and IL-28RA formation.^84^, ^85^, ^86^, ^87^ IFN-λ has the highest expression level among all the IFNs, and is secreted in mucosal barriers (like the respiratory tract) in response to viral infections. The target cells of IFN-λ3 include keratinocytes, neutrophils, macrophages, DCs, endothelial cells (ECs), and respiratory epithelial cells.^85^^,^^88^

IFN-λ can delay or prolong the JAK/STAT signaling pathway. Moreover, it can also initiate the mitogen-activated protein kinases (MAPK) and PI3-kinase pathways.^35^^,^^37^

IFN-λ or IFN-γ can bind to the type III IFN receptor (IFNLR). These receptors are mainly found on epithelial cells of many organ systems, including the respiratory, gastrointestinal, and reproductive system, and some myeloid cells. Type II alveolar epithelial cells produce IFN-λ to protect respiratory tract epithelium by the antiviral activity of this IFN. IFN-λ could be a promising therapeutic agent to reduce viral pathogenesis and the cytokine storm to prevent pneumonia and ARDS caused by COVID-19.^89^, ^90^, ^91^

It was reported that MERS-CoV protein functions as an IFN antagonist and suppresses IFN-λ production. During infection by SARS-CoV-2, IFN-λ reduced the viral load and inflammatory responses but had no effect on mortality rates.^92^^,^^93^

### Hematopoietic growth factors

#### Granulocyte colony-stimulating factors

Granulocyte colony-stimulating factors (G-CSFs) are glycoproteins that stimulate the production of granulocytes. They promote survival and proliferation of neutrophil precursors, and the function of mature neutrophils. G-CSFs can increase the production of ILs such as IL-10, IL-8, IL-6, and IL-1 and soluble tumor necrosis factor receptors (sTNF-Rs), and they also decrease the concentrations of IFN-λ, TNF-α, and granulocyte-macrophage colony-stimulating factor (GM-CSF).^89^ In the severe form of COVID-19, the concentrations of IL-2, IL-6, IL-10, and IFN-γ were elevated. Therefore, G-CSFs have been suggested to play a role in disease severity by facilitating the formation of the cytokine storm.^90^

Furthermore, G-CSFs may increase the oxidative burst within alveolar neutrophils and macrophages, which could increase the risk of respiratory failure during G-CSF administration. Cancer patients often receive G-CSFs to improve neutropenia caused by their cancer treatment. Therefore, the administration of G-CSFs in cancer patients with COVID-19 should be treated with caution.^91^^,^^94^

### GM-CSFs

GM-CSFs are important myelopoietic growth factors secreted by various cells such as epithelial cells and leukocytes. GM-CSF is an important pro-inflammatory cytokine stimulating both innate and adaptive immunity. There were increased numbers of GM-CSF-expressing leukocytes in the blood of COVID-19 patients. It was also reported that levels of GM-CSF were increased in COVID-19 patients compared with non-infected individuals.^3^^,^^95^

GM-CSF is probably implicated in the occurrence of the cytokine storm. It stimulates IL-1 and IL-6 production and targets macrophages and neutrophils. IL-1 and IL-6 play an important role in the cytokine storm, and they may also be aggravated by the cytokine storm itself.^96^

In COVID-19 patients suffering from life-threatening pneumonia, GM-CSF inhibition resulted in more rapid clinical improvement in symptoms and a further decrease in inflammation.^97^ By contrast, another study reported that GM-CSF had an effective role in macrophage homeostasis and pathogen clearance in the lungs.^98^ Due to these contradictory results, further research is needed to evaluate the effects of GM-CSF in COVID-19.

### TNFs

TNFs are pro-inflammatory cytokines significantly implicated in the cytokine storm. Levels of TNFs are high in COVID-19, and it has been suggested that early levels of TNFs can predict the mortality risk. Anti-TNF administration reduces the generation of other pro-inflammatory cytokines (like IL-1 and IL-6) in patients with rheumatoid arthritis. Thus, blocking TNF in individuals with COVID-19 is a potential immunomodulatory treatment by decreasing the production of other pathogenic cytokines. A major cause of lung failure in COVID-19 infection is capillary leakage, resulting from pro-inflammatory cytokines like TNF, IL-1, and IL-6. It has been suggested that anti-TNF therapy might reduce inflammation-mediated vessel leakage in COVID-19, but this requires further study.^99^^,^^100^

### TGF-β

TGF-β is well-known as a multifunctional immunoregulatory cytokine. It belongs to the TGF family. TGF-β is a potent immunosuppressive factor in the body that inhibits the immune response but accelerates healing. TGF-β may also reduce the number of lymphocytes in the peripheral circulation by inhibiting proliferation and differentiation.^101^ TGF signaling is activated via both canonical and non-canonical signaling pathways. A nucleocapsid protein interacting with Smad3 activates the canonical pathway, while a papain-like protein activates the non-canonical pathway, upregulating TGF-β. Apoptosis, inflammation, and fibrosis are all triggered by this activation, leading to lung failure and tissue loss. According to recent research, the untimely production of TGF-β is a feature of severe COVID-19 and may impair NK cell activity and early viral control.

Furthermore, SARS-CoV-2 has been shown to cause a TGF-β-related immune response that is not directed toward itself.^102^, ^103^, ^104^, ^105^ TGF-β inhibits the anti-oxidant system, activates the plasminogen activator inhibitor 1 (PAI-1) and nuclear factor-kappa-light-chain-enhancer of activated B cells, and downregulates the cystic fibrosis transmembrane conductance regulator (CFTR) via internalizing the epithelial sodium channel (ENaC) (NF-κB). These alterations result in inflammation, lung damage, and coagulopathy.^106^ Because TGF-β plays such an essential role in the initiation and development of the disease, several pieces of research have been conducted to target it for COVID-19 therapy.^107^, ^108^, ^109^, ^110^

TGF induction has been demonstrated to contribute considerably to the short- and long-term effects of COVID-19, while ACE inhibition affects TGF receptors, resulting in slight blunting of TGF-β receptor activity and effects.^111^^,^^112^ TGF- β1 levels in the blood were considerably higher throughout the early and middle phases of COVID-19 and were associated with SARS-CoV-2-specific IgA levels. As a result, the TGF-β1-IgA axis may play a key role in COVID-19 pathogenesis.^113^

### Chemokines

Chemokines aid in the fight against viral infections by attracting innate and adaptive immune cells to infection sites and boosting their cytotoxic action and production of antiviral mediators. On the other hand, some viruses employ chemokines to avoid the immune system. For example, certain large DNA viruses, such as herpesviruses, create chemokine-like molecules that disrupt signaling and the immune response, resulting in viral multiplication and persistence.^114^ Chemokines are further divided into four subtypes based on their main amino acid sequence: CC, CXC, CX3C, and XC. Atypical chemokine receptors (aCKRs) and conventional chemokine receptors (cCKRs) are the two types of chemokine receptors found on the surface of cells. Chemokine receptors may be found on a wide range of cells, including leukocytes, airway epithelial cells, mesenchymal cells, and ECs.^115^^,^^116^

Chemokines can help leukocytes to migrate, multiply, and degranulate. They can also increase cytokine production. Several chemokines have been found to have direct antibacterial activity.^115^^,^^117^ They have antiviral effects through several mechanisms. Chemokines direct cells from the innate and adaptive immune system to infection sites. Here they boost antiviral activity by promoting the generation of antiviral mediators.^118^ Compared with SARS and MERS, the high transmissibility and low fatality rate of SARS-CoV-2 might be attributed to distinct chemokine profiles, requiring more investigation.

CCL-2, CCL-7 (MCP-3), CXCL-9 (MIG), CXCL-10, and CCL-3 serum levels were shown to be higher in COVID-19 patients with clinical symptoms, according to Chi et al.^119^ COVID-19 patients have been shown to have higher levels of chemokines such as CCL3, CCL5, CXCL10, CCL19, CCL20, while CCL5 remained unchanged. When compared with healthy controls, Huang et al. found that the chemokines CXCL-8 (IL-8), CXCL-10 (IP-10), CCL-2 (MCP-1), CCL-3 (M1α), and CCL-4 (MIP-1β) had already reached high plasma levels, as well as elevated levels of several inflammatory cytokines (IL-IL-1α, IL-7, IL-9, IL-10, G-CSF, FGF, GM-CSF, PDGF, IFN-γ, TNF-α, and vascular endothelial growth factors (VEGFs).^95^

According to reports, T cells, NK cells, monocytes, macrophages, and neutrophils are the primary immune cells that invade the lungs in patients with COVID-19. CCL2, CCL7, and CCL8 are abundant in infiltrating macrophages at first.^120^, ^121^, ^122^ Corticosteroids have been found to reduce chemokines such as CXCL10 and CXCL8 that directly contribute to the pathogenesis of SARS-CoV-2.^123^ CCL2, CXCL10, and CXCL8 are the most commonly found chemokines associated with COVID-19 severity. Future research should to be focused on these cytokines. Furthermore, COVID-19 patients have higher CXCL-1, CXCL-2, and CXCL-6 expression than healthy people.^124^

### ILs

ILs are expressed by many types of cells, especially leukocytes. They play both pro-inflammatory and anti-inflammatory roles. ILs are involved in intercellular communication, activation, and migration of immune cells and cytokine production. They also mediate the proliferation, maturation, and adhesion of immune cells. There is a growing list of identified ILs, and approximately 40 different types of ILs are now known.^125^^,^^126^ Studies have observed abnormal levels of ILs in COVID-19 patients, such as IL-17, IL-13, IL-12, IL-10, IL-7, IL-6, IL-4, IL-2, and IL-1.^95^^,^^127^ The following is an overview of COVID-19-related ILs.

### IL-1

The prototypic pro-inflammatory cytokine IL-1 can be either IL-1α or IL-1β, whose bioactivities are indistinguishable. Some studies reported that IL-1α is produced by damaged ECs, epithelial cells, and myeloid cells. In comparison, IL-1β is released by infiltrating neutrophils and some monocyte-derived cells.^128^^,^^129^

The SARS-CoV-2 present in the lower respiratory tract can cause SARS associated with excessive pro-inflammatory cytokine levels like IL-1β. Anakinra is an antagonist for the IL-1 receptor capable of blocking IL-1α and IL-1β activity, and is used to manage auto-inflammatory conditions. One study found that using an IL-1 blocker ameliorated clinical symptoms in 72% of patients with COVID-19 and managed the ARDS.^130^^,^^131^

### IL-2

In 1976, IL-2 was described as a T cell growth factor (TCGF) during basic immunology research in human tumors. It can exert a pleiotropic effect on immunity and is routinely prescribed to manage some human cancers.^132^ It has been suggested that administration of IL-2 may be an effective therapy by increasing lymphocyte numbers in COVID-19 patients.^133^

IL-2 can stimulate all T cells, including T regulatory, T helper, and NK cells. These are vital antiviral cells essential for SARS-CoV-2 clearance. They can restrict the severe form of COVID-19-mediated cytokine storm. Therefore IL-2 has been suggested to be a therapeutic agent to control COVID-19.^134^^,^^135^

### IL-4

The anti-inflammatory cytokine IL-4 has a specific receptor (IL-4R). IL-4 can suppress the cytotoxic activity of macrophages and reduce nitric oxide (NO) production. Patients with COVID-19 have shown an elevated level of IL-4. IL-4 induces T cell differentiation into T helper 2 cells. These cells subsequently release additional IL-4 from other T cells in a positive-feedback loop. T helper 2 cells were found to be present in high amounts in COVID-19 patients.^136^, ^137^, ^138^, ^139^

### IL-6

The anti-inflammatory and pro-inflammatory cytokine IL-6 is expressed in various cells like ECs, T cells, and macrophages. The functions of IL-6 include differentiation of B cells resulting in IgM, IgE, and IgA production; regulation of T cell activation; and T cell differentiation.^134^^,^^135^

COVID-19 infection increases the expression level of IL-6, especially in patients with lung damage and pulmonary inflammation. Overproduction of IL-6 is more significant compared with other cytokines in the severe form of COVID-19. IL-6 has a pivotal role in the occurrence of cytokine storm in COVID-19.^140^^,^^141^

Elevated IL-6 levels are correlated with a poor prognosis of COVID-19 patients. IL-6 is a key mediator governing the generation of T helper 17 cells.^30^ Therefore, the excessive IL-6 concentration in COVID-19 patients may be a reason for the increased number of activated T helper 17 cells seen in these individuals. Moreover, it has been reported that excessive IL-6 signaling leads to an increase in effector T cells along with increased vessel permeability. Therefore, inhibition of IL-6 could be helpful in COVID-19 treatment, which may be due to its ability to inhibit the cytokine storm.^122^^,^^142^^,^^143^

### IL-7

The pleiotropic cytokine IL-7 is an important mediator for lymphocyte survival and proliferation. Lymphopenia and T cell depletion in the spleen and other organs can occur in severe COVID-19 patients, which reduces the ability to fight against viruses. IL-7 administration can be safe for those with life-threatening COVID-19, and can restore the lymphocytes to a usual number, appearing to reverse a pathologic characteristic of COVID-19. The IL-7-mediated restoration of lymphocytes boosted the antiviral activity and reduced the viral load.^143^, ^144^, ^145^

### IL-8

The pro-inflammatory cytokine IL-8 plays a crucial role in neutrophil chemotaxis and activation during inflammation. IL-8 levels increase in SARS-CoV-2, suggesting that IL-8 may contribute to neutrophilia in COVID-19 pathophysiology. It has been reported that IL-8 and IL-6 are closely correlated with COVID-19 severity (moderate to end-organ damage). The IL-8 concentration can make a distinct difference between severe progression and recovery in SARS-CoV-2 infection, and IL-8 could be a more appropriate marker to determine the status of COVID-19 compared with IL-6. Moreover, IL-8 could be a potential target in the treatment of COVID-19.^99^^,^^146^^,^^147^

### IL-10

IL-10 is usually considered to be an anti-inflammatory or immunosuppressive cytokine. It has been called cytokine synthesis inhibitory factor (CSIF).^140^

A particular property of the COVID-19-induced cytokine storm compared with that found in SARS-CoV infection is a significant increase in IL-10 in critically ill patients. A high concentration of IL-10 may be a predictor of a poor prognosis in COVID-19 cases. Moreover, the elevated concentration of IL-10 occurs earlier in the course of the infection compared with IL-6, and it was shown that IL-10 levels in serum were dramatically higher in COVID-19 patients admitted to the intensive care unit (ICU).^95^^,^^141^^,^^148^

Some studies have suggested that IL-10 acts as an anti-inflammatory cytokine by a negative feedback mechanism. At the same time, other clinical evidence has suggested that early elevation of IL-10 may have a detrimental role in COVID-19 intensity.^149^ Therefore more studies are needed on the role of IL-10.

### IL-17

IL-17 is an important mediator in innate immunity and an essential link between T cells and neutrophils.^150^ Moreover, in the ARDS inflammatory process, IL-17A (the most studied subtype of IL-17) acts on macrophages, resulting in increased expression of NO synthase, IL-1, IL-6, TNFα, and chemokines, which makes the disease worse. Moreover, alveolar endothelial and epithelial cells release IL-17, leading to the production of IL-6, GM-CSF, IL-1β, TNFα, TGF-β, and G-CSF from various cell types.^151^^,^^152^

It has been reported that IL-17 is involved in the hyperinflammatory state in COVID-19. The upregulation of IL-17A in the cytokine storm is produced by T helper 17 cells, and is mainly responsible for ARDS. Therefore, the possible therapeutic use of IL-17 inhibitors in COVID-19 has been suggested.^153^^,^^154^

### IL-21

Activated CD4+ T cells, NK T cells, and T helper 17 cells all release IL-21, which belongs to the γ-chain cytokine family. Both innate and adaptive immune responses are governed by IL-21. IL-21 promotes IgG1 and IgG3 class switching in humans while suppressing IgE class switching. Dendritic cells are activated by IL-21, which causes NK cells to release more IFN-γ, boosting their cytotoxic activity. IL-21 suppresses T helper 1 cell differentiation and promotes CD8+ T cell proliferation. IL-21 regulates the expression of CD27 and CD28, which is important for virus-specific effector CD8+ T cells.

In COVID-19 patients, measurement of serum IL-21 shows a high predictive value for disease development. High levels of IL-6 and IL-21 in the blood at admission are independent risk factors for clinical deterioration.^155^^,^^156^ IL-21 has also been demonstrated to reduce the generation of IL-6 and TNF-α, lowering the inflammatory proteins implicated in the cytokine storm.^157^ T follicular helper cell-induced IL-21 modulates germinal center B cell differentiation, which is critical for forming effective virus-neutralizing antibodies, similar to other viral infections. When the B cells in the germinal center of patients with acute COVID-19 were analyzed, IgG2-producing B cells responsive to type 1 IFN were dominant at the beginning of ICU hospitalization; however, as the disease progressed, the number of IgG1- and IgA1-producing B cells responsive to IL-21 and TGF-β increased, and ultimately the number of IgA2-producing B cells responsive to TGF-β increased. TGF-β produces lung fibrosis, the most preventable consequence of SARS-CoV-2 infection in the chronic post-inflammatory phase. In acute COVID-19, IL-21 and TGF-β may be out of balance, thus exacerbating the pathological state.^156^^,^^158^ IL-21 and IL-15 combined together promote a more effective immune response than either IL alone. As a result, a clinical trial exploring the combination of IL-15 and IL-21 for COVID-19 patients is recommended.^159^ However, anticancer clinical trials of IL-21 have been unsatisfactory, and it has been suggested that its clinical use is problematic.^156^ However, some research suggests that high circulating levels of IL-21 are linked to illness severity in COVID-19 patients.^160^

### IL-23

IL-23 belongs to the family IL-12 cytokine as a pro-inflammatory heterodimeric cytokine mainly secreted by the skin, intestinal mucosa, lung-activated macrophages, and DCs. IL-23 triggers IFN-γ expression and T cell proliferation, as well as IFN-α responsiveness to eliminate hepatitis C virus (HCV). It also enhances the resistance of the host to viruses by the IL-23/IL-17 axis. IL-23 triggers the production of suppressor of cytokine signaling 1 (SOCS1) and impairs T cell function in HIV infection. Biologics that target IL-23 are helpful for psoriasis treatment.^161^^,^^162^

It has been shown that low levels of IL-23 may lead to deficient immunity in the mucosal barrier, and an enhanced risk of respiratory infections.^161^ A study found that psoriasis patients treated with biologics had a higher risk for SARS-CoV-2 infection and hospital admission. However, there was no difference in mortality rate or mechanical ventilation in the ICU. At the same time, a case report study showed an improvement in SARS-CoV-2 infection after administration of guselkumab, an IL-23 antagonist.^163^^,^^164^

### IL-27

IL-27 is a pleiotropic cytokine and forms heterodimeric complexes of EBI3 and p28 subunits. IL-27 is involved in T helper 1 differentiation and acts on various cells, such as monocyte-derived, NK, T, and B cells. It is secreted by diverse cell types, especially activated macrophages and DCs. IL-27 signaling downregulates IL-4 and reduces mucin production. IL-27 can also induce anti-inflammatory responses by stimulating IL-10 production. It has been suggested that low levels of IL-27 could be a promising biomarker for COVID-19 prognosis.^164^, ^165^, ^166^

### IL-33

IL-33 belongs to the family of IL-1 cytokine with pleiotropic functions. IL-33 is produced by barrier tissues, and in the lungs damaged alveolar epithelial cells release IL-33.^167^ Reportedly, the production of IL-33 is associated with SARS-CoV-2 infection. IL-33 may be involved in pulmonary fibrosis by stimulating various cytokines. Anti-IL-33 therapy might be promising in COVID-19 management, and is presently under investigation.^168^

### Cytokine storm

**Figure 1:**
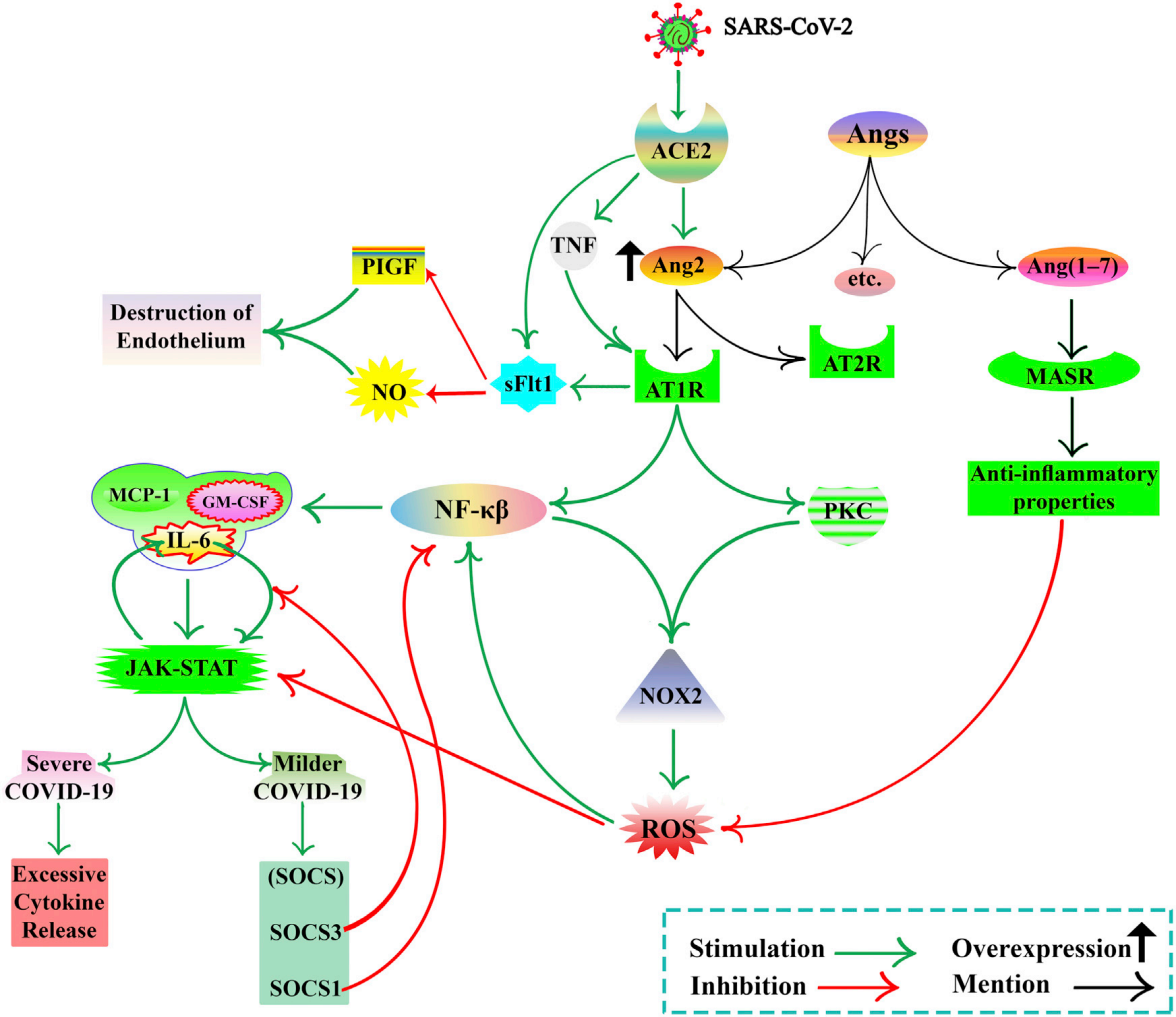
COVID-19 and cytokine storm, focusing on Ang2 concentration The concentrations of TNF-α, IL-6, and IL-10 are the most important mediator in cytokine storm formation. IL-6 receptors classify into two groups, mIL-6R and sIL-6R. During virus infection, host cells can express PRRs. PRRs detect PAMPs. ACE2 receptor acts as a PRR. ACE2 cleaves Ang2. ACE2 binds to viral S protein, so Ang2 concentrations increase. Ang2 has two types of receptors, AT1R and AT2R. The Ang2/AT1R complex activates PKC and NF-κB, leading to NOX2 activation and cytokine production. ROS production mediated by NOX2 and ROS activates NF-κB. NF-κB enhances the expression of IL-6, GM-CSF, MCP-1, etc. IL-6 induces the activation of NOX. IL-6, GM-CSF, and MCP-1 activate JAK-STAT signaling, resulting in an elevated level of SOCS, but, in severe disease, it leads to excessive cytokine production. SOCS blocks the JAK-STAT signaling. SOCS may provide a novel therapy for the treatment of COVID-19. The JAK-STAT pathway activates by ROS. IL-6 and JAK-STAT signaling pathway interaction can be defined as positive feedback. SOCS3 disrupts this vicious cycle by inhibiting IL-6 signaling. SOCS-1 inhibits NF-κB activation. NF-κB increases ACE2 expression. sFLT1 production is not clear (by binding the Ang2 to AT1 or directly induced by SARS-CoV-2 infection or upregulation of AT1 Receptor by TNF). sFLT1 inhibits PlGF, a VEGF, and impairs NO production, resulting in endothelial damage. Ang-(1–7) binds to the MASR and causes inhibition of ROS and anti-inflammatory properties. SARS-CoV-2, severe acute respiratory syndrome coronavirus 2; COVID-19, coronavirus disease 2019; Ang2, angiotensin2; IL, interleukin; TNF-α, tumor necrosis factor alpha; PRRs, pattern recognition receptors; ; PAMPs, pathogen-associated molecular patterns; ACE2, angiotensin-converting enzyme 2; AT1R, angiotensin II type 1 receptor; NF-κB, nuclear factor kappa-light-chain-enhancer of activated B cells; NOX, nicotinamide adenine dinucleotide phosphate oxidase; AT2R, angiotensin II type 2 receptor; GM-CSF, granulocyte-macrophage colony-stimulating factor; PKC, protein kinase C; ROS, reactive oxygen species; MCP-1, monocyte chemoattractant protein-1; SOCS, suppressor of cytokine signaling; JAK-STAT, Janus kinase-signal transducer and activator of transcription; sFLT1, soluble Fms-like tyrosine kinase-1; MASR, Mas oncogene receptor; gp130, glycoprotein 130; PlGF, placenta growth factor; NO, nitric oxide; sIL-6R, soluble IL-6 receptor; VEGF, vascular endothelial growth factor.

**Figure 2:**
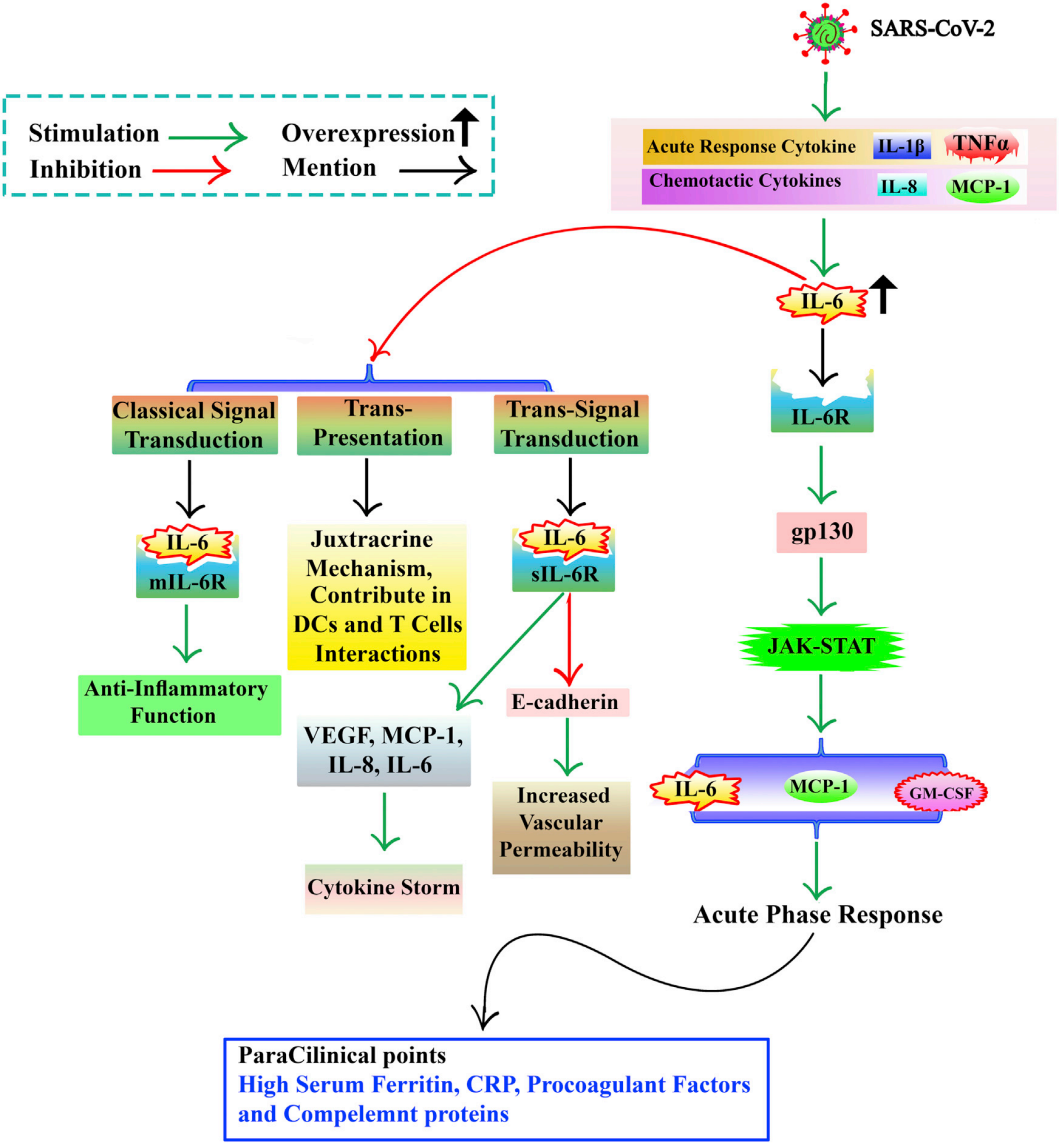
COVID-19 and cytokine storm, focusing on IL-6 IL-1b and TNF, as acute-response cytokines, and MCP-1 and IL-8, chemotactic cytokines, increase hypercytokinemia, which elevates IL-6. The IL-6/IL-6R acts on gp130 to increase IL-6, MCP-1, and GM-CSF by activating the JAK-STAT pathway. IL-6, GM-CSF, and MCP-1 may activate an acute-phase response indicated by a high serum ferritin, CRP, and pro-coagulant factors in paraclinical tests. Three IL-6 signal transductions of trans-presentation, trans-signal transduction, and classical signal transduction. IL-6/mIL-6R complex contributes to the classical signal transduction mode, which mediates anti-inflammatory function. IL-6 trans-signaling is more related to inflammatory processes. In this signaling, IL-6 binds to the sIL-6R. This leads to the production of VEGF, MCP-1, IL-8, IL-6, and E-cadherin. Expression in ECs is reduced. This increases vascular permeability and exacerbates the cytokine storm. IL-6 trans-presentation signaling pathway is a juxtracrine mechanism that contributes to dendritic and T cell interactions. mIL-6R, membrane-bound form of Interleukin-6 Receptor; CRP, C-reactive protein.

The role of the cytokine storm is illustrated in Figures 1 and 2. The cytokine storm, also called hypercytokinemia, is mostly an uncontrolled hyperimmune response to infection. This term was first introduced in 1993 with regard to tissue graft rejection. Since then, the term cytokine storm has been more often used in viral infections, such as influenza viruses, SARS, and MERS. Studies have found that in SARS, high concentrations of pro-inflammatory cytokines, such as TNF-α, IL-6, IL-1β, IL-12, IFN-γ, IP10, and MCP1, were associated with lung damage.^169^^,^^170^ The cytokine storm has also been observed in some COVID-19 patients. Extraordinarily high levels of inflammatory cytokines were found in life-threatening and severe forms of COVID-19, resulting in pulmonary inflammation, lung failure, and MODS. It was also reported that the levels of IL-1, TNF-α, and IL-6 could clearly differentiate mild cases from severe forms of COVID-19.^95^

The concentrations of IL-6, TNF-α, and IL-10 were reported to be related to the severity of SARS-CoV-2 infection. Raised concentrations of IL-1β, IFN-γ, MCP1, and IP10 in ICU COVID-19 patients might stimulate the activation of T helper 1 cells to further increase inflammation.^95^^,^^170^

IL-6 is one of the most important mediators in the cytokine storm. Various cells like T cells, ECs, fibroblasts, and monocyte-derived cells secrete IL-6, which then acts on B cells, T cells, and granulocytes.^134^ IL-6 receptors can be classified into two groups: soluble IL-6 receptor (sIL-6R) and membrane-bound IL-6 receptor (mIL-6R). mIL-6R is expressed by only some of the target cells of IL-6. sIL-6R functions as a transporter of IL-6 to various parts of the body, and is generated by mIL-6R cleavage mediated by a disintegrin and metallopeptidase domain 10 (ADAM10) and ADAM17. It has also been reported that sIL-6R can be produced by alternative splicing of IL-6R mRNA.^134^

During virus infection, host cells can express pattern recognition receptors (PRRs) on their surface. PRRs detect molecules related to pathogens (bacteria, fungi, and viruses) that are different from host molecules, called pathogen-associated molecular patterns (PAMPs). The ACE2 receptor recognized by SARS-CoV-2 serves as a PRR expressed in many types of cells, such as pulmonary alveolar cells and ECs in blood vessels.^171^

ACE2 is a zinc metalloprotease enzyme that cleaves angiotensin II (Ang2). Peptides that are produced as a result of Ang2 cleavage may activate signaling pathways that counteract Ang2 signaling. ACE2 binds to the viral S protein so its activity is reduced; therefore, Ang2 concentrations may be significantly higher in infected lungs.^172^

Ang2 type 1 receptor (AT1R) is considered an important Ang2 receptor. The Ang2/AT1R complex can activate several molecules and signaling pathways in ECs, such as protein kinase C (PKC) and NF-κB. NF-κB acts as a transcription factor leading to NADPH oxidase (NOX) 2 activation, cytokine production, etc. Reactive oxygen species (ROS) production in ECs is increased by NOX2, and the ROS can activate the NF-κB signaling pathway.^173^^,^^174^

Activation of NF-κB enhances the expression of many cytokines, such as IL-1, TNF-α, IL-6, IL-2, IL-12, GM-CSF, IL-8, MCP-1, and MIP-1. Moreover, IL-6 can induce the activation of NOX, resulting in oxidative stress.^167^^,^^175^^,^^176^

The main source of MCP-1, GM-CSF, and IL-6 are fibroblasts, ECs, epithelial cells, etc. These cytokines may affect macrophages, neutrophils, and other immune cells via the JAK-STAT signaling pathway resulting in increased levels of suppressor of cytokine signaling (SOCS), and excessive cytokine production in severe COVID-19.^177^, ^178^, ^179^, ^180^

The SOCS family function as intracellular checkpoint inhibitors. SOCS regulates JAK-STAT signaling in a negative feedback loop. STATs enhance transcription of the SOCS genes, while SOCS proteins bind to JAKs to inhibit the JAK/STAT pathway. The JAK/STAT pathway is critically involved in the transmission of extracellular cytokine signals into the nucleus.^181^ Therefore, SOCS may be a target for the treatment of COVID-19, using SOCS mimetics or stabilizers. The JAK-STAT pathway can be inhibited by SOCS, and activated by ROS.^182^^,^^183^

IL-6 activates STAT3, and to a lesser extent STAT1. The JAK-STAT pathway enhances the generation of IL-6 in a positive-feedback loop. SOCS3 could disrupt this vicious cycle in severe COVID-19 by inhibiting IL-6 signaling.^184^^,^^185^

SOCS can also interfere with NF-κB activity. It has been reported that, in infected cells, inhibition of SOCS1 increases the IκB protein concentration, and dramatically increases NF-κB activation. NF-κB can modulate ACE2 expression, and one study reported that ACE2 mRNA could be suppressed by NF-κB inhibitors, such as pyrrolidine dithiocarbamate (PDTC).^186^^,^^187^

Since SARS-CoV-2 can bind to ACE2, NF-κB inhibitors have been suggested to be beneficial in COVID-19. This binding also decreases the level of ACE2, resulting in increased Ang2 levels. The increased Ang2 binds to its receptors (AT1 and AT2) and directly impairs endothelial function.^188^

Soluble fms-like tyrosine kinase-1 (sFLT1) is a soluble inhibitor of VEGFs. It acts as an endothelial decoy receptor produced by binding of Ang2 to AT1 in response to hypoxia, only in ECs or monocytes. It was shown that the level of sFLT-1 was considerably higher in COVID-19 patients with pneumonia.^189^, ^190^, ^191^

It has not yet been determined whether sFLT-1 production is directly triggered by SARS-CoV-2 infection or stimulated in response to increased Ang2 concentrations. There is also a hypothesis that COVID-associated inflammatory mediators directly or indirectly cause the production of sFLT-1; for example, TNF can induce AT1 receptor upregulation.^191^^,^^192^

sFLT1 has many functions, such as inhibition of VEGFs, such as placental growth factor (PlGF); impairment of NO production resulting in endothelial damage; and sensitizing ECs to Ang2 resulting in the formation of a positive-feedback loop. It has also been reported that the Ang2/AT1 complex induces cytokine production and ROS generation via the NF-κB pathway in macrophages.^177^^,^^193^^,^^194^

In addition to Ang2, there are other biologically active members of the angiotensin peptide family. Some studies have been performed on the role of Ang-(1–7) in COVID-19 disease. However, no study has been done on other types of angiotensins in SARS-CoV-2 infection. Ang-(1–7) binds to the MAS1 oncogene receptor (MASR), a G protein-coupled receptor. This inhibits ROS production and vasodilation, and has anti-inflammatory properties. Therefore Ang-(1–7) may be beneficial in COVID-19.^195^

It has been reported that the levels of TNF and IL-1 (acute-response cytokines) as well as MCP-1 and IL-8 (chemotactic cytokines) increase initially in hypercytokinemia, resulting in a sustained increases in IL-6 concentration. The IL-6/IL-6R axis acts on transmembrane glycoprotein 130 (gp130) to increase the level of IL-6 in a positive-feedback loop. GM-CSF and MCP-1 activate the JAK-STAT pathway to prolong the inflammatory process.^178^^,^^179^

IL-6 and other cytokines activate an acute-phase response. Acute-phase proteins are stimulated by IL-6 and secreted from the liver, including C-reactive protein (CRP), ferritin, pro-coagulant factors like fibrinogen, serum amyloid A (SAA), haptoglobin, and a1-antichymotrypsin. At the same time, the levels of fibronectin, albumin, and transferrin are reduced due to IL-6 activation. High serum ferritin levels, CRP, and pro-coagulant factors can be measured by paraclinical tests.^180^

GM-CSF and possibly IL-6 are secreted by the pathogenic IFN-ɣ+ GM-CSF+ T helper 1 cells to increase inflammatory CD14+CD16+ monocyte responses by increasing the concentration of IL-6 in COVID-19 patients. Moreover, monocytes secrete TNF-α, IL-6, and IL-1b.^3^

IL-6 employs a highly complex signaling pathway, making it difficult to know exactly how IL-6 signaling exerts its biological effects. There are three main IL-6 signal transduction mechanisms: trans-presentation, trans-signal transduction, and classical signal transduction. IL-6 receptor signaling has protective roles in normal individuals, such as regulating various metabolic processes and tissue repair.^196^

It has been reported that the IL-6/mIL-6R complex contributes to the classical signal transduction process, which mediates the anti-inflammatory activity of IL-6. This complex causes gp130 dimerization, since gp130 is expressed by all cells. Studies have suggested that IL-6 can bind to both forms of the receptor and subsequently interact with gp130. Activated gp130 initiates intracellular signaling pathways that finally alter gene expression. IL-6 signal transduction by gp130 activation in cells that do not express mIL-6R can also be triggered by sIL-6R binding. Therefore, sIL-6R increases the target cells that can respond to IL-6 and accounts for its pleiotropic functions.^196^, ^197^, ^198^

IL-6 trans-signaling is more related to pro-inflammatory processes. In this signaling, IL-6 binds to sIL-6R, and then a gp130 dimer complex is formed. Next, there is activation of IL-6-sIL-6R-JAK-STAT3 signaling. Finally, this causes the production of VEGF, IL-8, MCP-1, and additional amounts of IL-6. Moreover, the production of E-cadherin in ECs is reduced, thereby increasing vascular permeability and exacerbating the cytokine storm.^143^^,^^198^^,^^199^

The IL-6 signaling pathway of trans-presentation is a juxtracrine mechanism that contributes to DC and T cell interactions. It may lead to the stimulation of pathogenic T helper 17 cell production, inhibition of T regulatory cells, and inflammation.^199^

The COVID-19-induced cytokine storm mechanism is complex, and many molecules and cells in various cascades and feedback loops are involved in creating it or eliminating it. Therefore, there is a need for further research to reach a more solid understanding of this condition, because the early diagnosis of cytokine storms is important to decrease the mortality rate of COVID-19.

### Molecular mechanisms of cytokine production in SARS-CoV-2

During SARS-CoV-2 infection, the immune response is divided into a protective phase based on immunological defense against the virus, and a second phase marked by severe inflammation. Because the virus inhibits the host immune response in the first stage and causes an inflammatory storm in the second stage, some SARS-CoV-2-infected patients become seriously ill. DCs, neutrophils, macrophages, and NK cells are the first line of defense in the immune, and influence the type and severity of the response. The SARS-CoV-2 cytokine storm is fueled by these cells and other tissue-resident cells, including epithelial cells and ECs. COVID-19 patients have high levels of pro-inflammatory cytokines and chemokines such as IL-1β, IL-2, IL-6, IL-7, IL-10, IFN-γ, TNF-α, G-CSF, CCL2, and CXCL10. These inflammatory chemokines and cytokines then recruit additional innate immune cells from the peripheral tissues and activate adaptive immune cells (CD4+ and CD8+ T cells) to produce long-lasting inflammatory cytokines like IL-2, IFN-γ, and TNF-α, which cause myelopoiesis, leading to rapid granulopoiesis, and aggravate lung epithelial damage. Furthermore, macrophage activation is triggered by the overexpression of systemic cytokines, including IL-2, IFN-γ, GM-CSF, and TNF-α. These cytokines eventually result in ARDS. As a result, SARS-CoV-2 infection causes an overproduction of pro-inflammatory cytokines, often resulting in severe consequences.^200^^,^^201^

The heterodimeric inhibitory receptor CD94/NK group 2 member A (NKG2A) is expressed by NK cells. NKG2A can bind to peptide-loaded non-classical human leukocyte antigen (HLA) class I molecules (HLA-E) to reduce NK cell toxicity and cytokine release. NKG2A expression was found to be high in COVID-19 patients in some studies.^202^^,^^203^ Due to SP1 intracellular expression in lung epithelial cells, NK cells displayed elevated NKG2A/CD94 inhibitory receptor levels, according to a recent study.^204^

One recently discovered beta CoV protein is SARS-CoV-2 ORF8. ORF8 is a hypervariable gene that allows the virus to adapt to the human host more easily.^205^ ORF8 was identified as the most critical viral protein that was inhibited by type I IFN (IFN-β) and NF-κB-responsive promoters. ORF8 also influenced intracellular immunity and growth pathways through IFN-I signaling and MAPKs.^206^

After recognizing a wide range of PAMPs via PRRs such as toll-like receptors (TLRs), macrophages generate pro-inflammatory cytokines. The SARS-CoV-2 spike protein S1 component substantially promotes IL-6 and IL-1β production in murine and human macrophages by activating TLR4 signaling via the JNK and NF-κB pathways, according to Shirato et al.^207^^,^^208^ In macrophages, the P44/42 MAPK and Akt pathways produce pro-inflammatory molecules. Infection with SARS-CoV-2 activated the JAK-STAT pathway via CD147, resulting in increased production of cyclophilin A (CyPA), which then bound to CD147 and activated the MAPK pathway. PAMPs initiate various signaling cascades in the innate immune system, leading to the transcriptional activation of type I and type III IFNs.^31^^,^^209^

### NSPs from SARS-CoV-2 have a role in immune regulation

The CoV NSPs are essential components of the viral replication mechanism. They aid viral RNA transcription and replication while evading the natural host defenses.^210^ The (alpha-beta)-CoV genome is a non-segmented, single-strand, positive-sense RNA that encodes for six primary ORFs. The first two-thirds of viral RNA contains two overlapping reading frames, ORF1a and ORF1b, which encode large polyproteins that are then cleaved into 16 mature NSPs. These are thought to play essential roles in regulating cellular pathways and adjusting the cellular environment for optimized virus replication while attempting to avoid immunity.^195^^,^^211^

The virus capacity to avoid and inhibit the host innate immune responses is one of the most critical drivers of viral infection. Both alpha and beta-CoV nsp1 proteins are likely to promote more effective, coordinated immune suppression. Nsp1 proteins have been demonstrated to interfere with multiple phases of the immune response.^195^ Different strains of (alpha-beta)-CoVs have been shown to suppress IFN-I with variable degrees of efficiency. Nsp1 and nsp6 effectively suppressed IFN-I via blocking STAT1 and STAT2 phosphorylation.^212^ IFN-β-expression was measured after Nsp1 transfection into HEK293T cells, and activity from a reporter plasmid expressing an IFN transcription factor in a study to examine the immune response effects of SARS-CoV nsp1. Nsp1 can suppress the activation of NF-κB, IRF3, IRF7, and the transcription factor c-Jun, according to the findings.^213^^,^^214^ SARS-CoV-2 nsp9 and nsp10 target NKRF (NF-κB repressor) to increase IL-6/IL-8 production, according to one study using peripheral blood mononuclear cells (PBMCs).^215^ Nsp3 is a multi-domain transmembrane protein containing an ADP-ribose phosphatase domain (ADRP/MacroD) that is thought to disrupt the host immune response by removing ADP-ribose from ADP-ribosylated proteins or RNA. The PLPro/deubiquitinase domain cleaves viral polyprotein and inhibits the host innate immune response.^216^, ^217^, ^218^ Furthermore, the papain-like proteinase nsp3 has been shown to reduce protective immunity by suppressing innate immunity genes and preventing the host immunological response.^219^

Another study found that SARS-CoV-2 nsp6 could disrupt lysosome acidification in lung epithelial cells by interacting with the vacuolar-type H+-ATPase component ATP6AP1, resulting in blockage of autophagic flux, pyroptosis, and activation of NLRP3 inflammasomes.^220^ It has been found that nsp6 can suppress IFN regulation by interacting with TANK binding kinase 1 (TBK1). Orf6 binds importin karyopherin α 2 (KPNA2) to restrict IRF3 nuclear translocation. Nsp13 binds and prevents TBK1 phosphorylation, while Orf13 binds and blocks IRF3 nuclear translocation.^221^ Another study found that nsp8 and nsp9 both bind to 7SL RNA in the signal recognition particle after infection, and prevent protein trafficking to the cell membrane. The IFN response to viral infection is suppressed when these essential cellular processes are disrupted.^222^ Xu et al. recently discovered that NSP12 might activate RIPK1 and cause a cytokine storm leading to death.^223^

### miRNAs involved with IFNs and ILs

miRNAs are a class of short (20–25 or 21–23 bases) single-stranded oligonucleotides, acting as non-coding RNAs (ncRNAs). It has been reported that the expression of one-third of all human genes can be regulated by miRNAs.^22^^,^^23^

miRNAs are produced from pri-miRNAs that then form pre-miRNAs with a stem-loop structure. Endonucleases process the pre-miRNAs to form mature miRNA duplexes. The miRNA duplex is finally loaded into the Argonaute protein, resulting in the formation of a mature RNA interference silencing complex (RISC).^224^ The mature single-stranded miRNA regulates gene expression in a post-transcriptional manner mediated by binding to sites within the 5′- or 3′-untranslated region of complementary messenger RNAs.^22^ Moreover, miRNAs may be involved in cell-to-cell signaling by entering into neighboring cells and affecting the expression of their mRNAs.^225^

miRNAs can affect many signaling pathways and regulate cytokine production. Moreover, cytokines are capable of altering miRNA expression by triggering transcription factor miRNAs. It is known that the miRNAs expression profile is specific to the type of cell.^226^^,^^227^

We have provided a list of cytokine-related miRNAs involved in COVID-19 pathogenesis. Understanding the miRNAs that regulate cytokines or are regulated by them can help us to better understand their role in the pathogenesis and clinical treatment of COVID-19, since miRNA expression is specific to the type of cell.^228^ We suggest that the expression profile of these cellular miRNAs in COVID-19 patients be examined to help identify the major cells involved in SARS-CoV-2 infection.

There have been numerous reports about IFN-related miRNAs (Table 1). Within the type 1 IFNs family, only IFN-α and IFN-β have been studied in depth. It was reported that IFN-α was regulated by miR-466l, miR-22, and miR-122,^229^, ^230^, ^231^ while miR-146a, miR-26a, miR-34, and Let-7b can regulate IFN-β.^232^^,^^233^ In addition, it was observed that IFN-α regulates the production of miR-130a/301, miR-203, and miR-122.^234^, ^235^, ^236^ Many types of miRNAs are known to be regulated by IFN-β. These are miR-155, miR-29a, miR-26a, miR-34a, Let-7b, miR-21, and miR-122.^233^^,^^234^^,^^237^, ^238^, ^239^ Moreover, it was shown that IFN-τ (not found in humans) can regulate bta-miR-204 in bovine endometrial epithelial cells.^240^

**Table 1:** IFNs

IFNs	Subtype	Cell source	Target cells	Common pathway	Known functions	COVID-19	Regulated miRNA	Regulatory miRNAs	References
IFN-I	IFN-α	pulmonary epithelial cells, DCs, macrophages	many cell types, B cells, and monocytes	NF-κB, JAK-STAT (MAPK, PI3-kinase)	induces ACE2 as an ISG in human upper airway epithelial cells	reduced the number of viruses, resulting in relief of symptoms, leading to shorter disease duration	miR-130a/301, miR-203, miR-122	miR-466l, miR-22, miR-122	34, 35, 36, 37, 38^,^^41^, ^42^, ^43^, ^44^, ^45^, ^46^^,^^156^, ^157^, ^158^^,^^161^, ^162^, ^163^^,^^241^
	IFN-β	pulmonary epithelial cells, DCs, macrophages	immune cells (B cells, T cells), monocytes, macrophages, DCs	NF-κB, JAK-STAT (MAPK, PI3-kinase)	effective antiviral action via ISGs	IFN-β-1b administration in severe COVID-19 had positive effects on clinical improvement and duration of hospital stay without serious adverse effects in patients	miR-155, miR-29a, miR-26a, miR-34a, Let-7b, miR-21, miR-122	miR-146a miR-26a miR-34, Let-7b	34, 35, 36, 37, 38^,^^41^^,^^47^, ^48^, ^49^, ^50^, ^51^, ^52^, ^53^, ^54^^,^^159^, ^160^, ^161^^,^^164^, ^165^, ^166^
	IFN-κ	macrophages, monocytes, DCs, keratinocytes	monocytes, dendritic cells	NF-κB, JAK-STAT (MAPK, PI3-kinase)	influence innate immune system cells. Improved symptoms such as cough in patients with asthma	IFN-κ plus TFF2 could significantly enhance clinical improvement	not reported	not reported	^31^^,^^34^, ^35^, ^36^, ^37^, ^38^^,^^55^, ^56^, ^57^
	IFN-δ	porcine blastocysts	not reported	not reported	antiviral and immunomodulatory activity. Lower antiviral activity than IFN-α	not reported	not reported	not reported	^58^^,^^59^
	IFN-ε	lung, brain, skin tissue, intestinal system, reproductive tissues (Uterus, Cervix, Vagina, Ovary)	macrophages	NF-κB, JAK-STAT (MAPK, PI3-kinase)	mucosal immunity against viral and bacterial infections. Suppression of HIV replication. Protection of reproductive system against viral infections	may be explanation for lower mortality rate in women with SARS-CoV-2 infection than men	not reported	not reported	34, 35, 36, 37, 38^,^^60^, ^61^, ^62^, ^63^, ^64^, ^65^, ^66^, ^67^, ^68^
	IFN-τ	bovine blastocysts, endometrial cells	can affect human macrophages	JAK-STAT (bovine)	reduced inflammatory cell infiltration into lung tissue in mouse model of allergy. Antiviral activity. Antiproliferative effects	not reported	bta-miR-204 (bovine endometrial epithelial cells)	not reported	69, 70, 71^,^^242^^,^^243^
	IFN-ω	dendritic cells	leukocytes, epithelial cells	NF-κB, JAK-STAT (MAPK, PI3-kinase, P13K/Akt) signaling)	antiviral effects	anti-SARS activity similar to IFN-β. Useful in severe COVID-19 patients with pneumonia	not reported	not reported	34, 35, 36, 37, 38^,^^72^, ^73^, ^74^, ^75^, ^76^
	IFN-ζ	in mice bronchial epithelial cells, salivary duct cells	IFN-α/βR-expressing cells	not exactly known (IRF-1 pathway?)	antiviral and immunomodulatory effects	not reported	not reported	not reported	^77^^,^^79^
IFN-II	–	bronchial epithelial cells, NK cells, T cells, macrophages	T cells, NK cells	NF-κB, JAK-STAT (MAPK, PI3-kinase)	inhibited acute inflammation (inhibited innate/acquired immunity transition)	expression of IFN tends to be lower in severe COVID-19 than mild cases	miR-29a, miR-155, miR-520b	miR-29, miR-181a	80, 81, 82, 83^,^^168^^,^^243^, ^244^, ^245^, ^246^, ^247^
IFN-III	IFN-λ1 (or IL-29), IFN-λ2 (or IL-28A), IFN-λ3 (or IL-28B), IFN-λ4	epithelial cells, macrophages, DCs, cytotoxic T cells, NK cells, regulatory T cells	keratinocytes, neutrophils, macrophages, DCs, ECs, respiratory epithelial cells	JAK-STAT (MAPK, PI3-kinase)	reduced systemic inflammation	reduced viral load and inflammatory responses	miR-15a	miR-548, miR-29	^35^^,^^37^^,^^84^, ^85^, ^86^, ^87^, ^88^^,^^92^^,^^93^^,^^169^^,^^248^, ^249^, ^250^, ^251^, ^252^

Some IFN-II- and IFN-III-related miRNAs have also been identified. It was observed that miR-29 and miRNA181a can regulate IFN-II,^240^^,^^253^ while miRNA-548 and miR-29 affect IFN-III.^254^^,^^255^ It was also reported that IFN-II affects some miRNAs, miR-29a, miR-155, and miR-520b,^256^, ^257^, ^258^, ^259^ while IFN-III can regulate miR-15a.^260^

In addition, the effects that ILs and miRNAs have on each other have been investigated (Table 2). The expression of many COVID-19-related ILs can be altered by miRNAs. For instance, IL-1 is affected by miRNA-146a;^261^ IL-2 by miRNA-221-3p;^262^ and IL-4 by miRNA-221-3p, miR-210, miR-524-5p, and miR-340/429.^263^, ^264^, ^265^, ^266^ IL-6 is affected by mmu-miR-7578, miRNA-136-5p, miRNA-146a, miRNA-30b, and miR-365;^267^, ^268^, ^269^, ^270^, ^271^ IL-8 by miRNA-146a, miR-520b, miR-155, miR-106a, and miR-16;^272^, ^273^, ^274^, ^275^, ^276^ IL-10 by miRNA 27a-3p, hsa-miR-106a, and hsa-miR-106a;^277^, ^278^, ^279^ IL-17 by miR-30a and miR-136;^280^^,^^281^ IL-23 by miRNA-155;^282^ and IL-33 by RNA-200, miR-524-5p, and miR-378a-3p.^283^, ^284^, ^285^

**Table 2:** ILs

IL type	COVID-19	Regulated miRNA	Regulatory miRNA	References
IL-1	IL-1 inhibitor improved clinical symptoms in 72% of patients with ARDS Excessive expression of pro-inflammatory cytokines like IL-1β in lower respiratory tract cells	miR-155	miR-146a	^118^^,^^119^^,^^170^^,^^194^
IL-2	administration of IL-2 may be effective by increasing lymphocyte numbers in critically ill patients Il-2 can stimulate T cells, including T helper, T regulatory, and NK cells, which are essential for viral clearance, and limiting the severe cytokine storm	not reported	miR-221-3p	^121^^,^^122^^,^^171^
IL-4	patients have elevated IL-4	miR-124, miR-142-5p, miR-130a-3p	miR-221-3p, miR-210, miR-524-5p, miR-340/429	^126^^,^^167^^,^^172^, ^173^, ^174^^,^^177^^,^^286^
IL-6	high IL-6 concentrations have been reported correlated with patient pulmonary inflammation and lung damage elevated level IL-6 is correlated with poor prognosis inhibition of IL-6 can be effective in blocking cytokine storm	miR-15a/-16, miR-1275	mmu-miR-7578, miR-136-5p, miR-146a, miR-30b, miR-365	^129^^,^^130^^,^^175^^,^^176^^,^^178^^,^^179^^,^^287^, ^288^, ^289^
IL-7	IL-7 administration was safe in life-threatening COVID-19. Restored lymphocytes to a normal count, reversed COVID-19 pathology	miR-6852	–	^132^^,^^133^^,^^180^^,^^290^
IL-8	IL-8 is a more appropriate marker than IL-6 potential therapeutic target	mir-200	miR-146a, miR-520b, miR-155, miR-106a, miR-16	^99^^,^^136^^,^^137^^,^^181^, ^182^, ^183^, ^184^^,^^196^^,^^291^
IL-10	IL-10 acts as an anti-inflammatory cytokine by a negative feedback mechanism. Other clinical evidence suggested that early IL-10 elevation may play a pathological role	miR-155, miR-375	miR-27a-3p, miR-106a, miR-106a	^134^^,^^185^, ^186^, ^187^^,^^196^, ^197^, ^198^
IL-17	IL-17 is related to hyperinflammatory state and cytokine storm upregulation of IL-17A produced by increased T helper 17 cells is partly responsible for ARDS Possible therapeutic use of IL-17 inhibitors	miR-873, miR-155-5p, miR-497	miR-30a, miR-136	141, 142, 143^,^^188^^,^^189^^,^^198^^,^^199^^,^^292^
IL-21	in acute COVID-19, TGF-β and IL-21 may be out of balance, exacerbating disease	miR-663b, miR-29	miR-155, miR-30b, miR-423-5p	244, 245, 246, 247^,^^251^^,^^252^
IL-23	IL-23 antagonist improved clinical symptoms	miR-25	miR-155	^147^^,^^190^^,^^199^
IL-27	IL-27 can induce anti-inflammatory effects by stimulating IL-10 production. Low levels of IL-27 may be a prognostic marker for COVID-19	miR-935	–	^140^^,^^147^^,^^148^^,^^200^
IL-33	IL-33 production was increased	miR-320	miR-200, miR-524-5p, miR-378a-3p	^150^^,^^191^, ^192^, ^193^^,^^201^

It has also been demonstrated that ILs can regulate the concentration of many miRNAs. For instance, IL-1 regulates miRNA-155;^293^ IL-4 regulates miR-124, miR-142-5p, and miR-130a-3p;^294^^,^^295^ IL-6 regulates miRNA-15a/-16 and miR-1275;^296^^,^^297^ IL-7 regulates hsa-miR-6852;^298^ IL-8 regulates mir-200;^299^ IL-10 regulates miR-155 and miR-375;^299^, ^300^, ^301^ IL-17 regulates miR-873 and miR-155-5p;^301^^,^^302^ IL-23 regulates miRNA-25;^302^ IL-27 regulates miR-935;^303^ and IL-33 regulates miR-320.^304^

There is a wide range of cytokine-related miRNAs that it is not possible to cover completely in this article. We suggest that future research should concentrate on those miRNAs that are associated with all COVID-19-related cytokines.

### Conclusion

The COVID-19-mediated cytokine storm involves an imbalance between anti-inflammatory cytokines and pro-inflammatory cytokines, leading to an overproduction of pro-inflammatory cytokines. This causes serious problems, including tissue damage, ARDS, MOD, and death. There are some clinical studies about the early diagnosis of cytokine storms. This is essential to decrease mortality. There are some discrepancies in published studies about the effects of various cytokines on the treatment of COVID-19, and some studies have suggested that the disease phase is very important in the clinical use of cytokines and that they will have different effects. Moreover, we suggest that cytokine-related miRNAs may be a promising new target for the treatment of COVID-19. Based on the data, Ang(1–7), MASR, SOCSs, NF-kβ inhibitors, and JAK-STAT inhibitors should be further investigated as potential therapeutic agents.

## Availability of data and material

The primary data for this study are available from the authors on request.
